# Differences between Advanced Large Cell Neuroendocrine Carcinoma and Advanced Small Cell Lung Cancer: A Propensity Score Matching Analysis

**DOI:** 10.7150/jca.84600

**Published:** 2023-05-21

**Authors:** Weichang Yang, Wenjun Wang, Zhouhua Li, Juan Wu, Xiaofeng Xu, Chong Chen, Xiaoqun Ye

**Affiliations:** Department of Respiratory and Critical Care Medicine, The Second Affiliated Hospital of Nanchang University, Nanchang University, Nanchang, Jiangxi, P.R. China.

**Keywords:** large cell neuroendocrine carcinoma, small cell lung cancer, advanced, propensity score matching analysis, SEER database.

## Abstract

**Background:** Nowadays, the characteristics and treatment of advanced pulmonary large cell neuroendocrine carcinoma (LCNEC) remain controversial. This study aimed to analyze the similarity of clinical characteristics, survival outcomes and treatment modalities between advanced LCNEC and advanced small cell lung cancer (SCLC) to provide more evidence for the study of advanced LCNEC.

**Methods:** All SCLC and LCNEC patient data were obtained from the SEER database (2010-2019). Pearson's χ2 test was used to compare the differences in clinical characteristics. Propensity score matching (PSM) was utilized to balance the bias of the variables between patients. Univariate and multivariate Cox proportional hazards regression analyses were performed to identify prognostic factors. KM analysis was used to calculate survival.

**Results:** A total of 1094 patients with IV LCNEC and 20939 patients with IV SCLC were included in this study. The demographic characteristics and tumor characteristics of IV LCNEC and IV SCLC were different (p < 0.05). After PSM, the overall survival (OS) for IV LCNEC and IV SCLC was 6.0 months, the cancer-specific survival (CSS) was 7.0 months, and there was no significant difference in OS or CSS between the two groups. Risk/protective factors for OS and CSS were similar for IV LCNEC and IV SCLC patients. Survival outcomes were similar in patients with IV LCNEC and IV SCLC with different treatment modalities; chemoradiotherapy significantly improved OS and CSS in patients with IV LCNEC (9.0 months) and SCLC (10.0 months), however, radiotherapy alone did not improve survival in patients with IV LCNEC.

**Conclusions:** These results confirmed that the prognosis and treatment modalities are similar and that advanced LCNEC could be treated as advanced SCLC, which provide new evidence for the treatment of advanced LCNEC patients.

## Introduction

Lung cancer is one of the most common tumors worldwide, increasing the global medical and economic burden 1. Pulmonary large cell neuroendocrine carcinoma (LCNEC) is a rare tissue type of lung cancer, accounting for approximately 3% of all types of lung cancer, and its characteristics have not received sufficient attention due to its rarity 2. LCNEC exhibits neuroendocrine characteristics, immunohistochemistry (IHC) features, biological markers and morphological characteristics, which are similar to small cell lung cancer (SCLC); therefore, the WHO classified LCNEC and SCLC as pulmonary high-grade neuroendocrine carcinoma (HGNEC) in 2015 3. Of note, due to the highly metastatic characteristics of SCLC and LCNEC, patients are often in an advanced stage of the tumor at the time of diagnosis, which increases the difficulty of treatment 4, 5.

The WHO reclassified LCNEC as pulmonary HGNEC, however, LCNEC is not fully equivalent to SCLC 6. Previous studies found that LCNEC has both SCLC and non-small cell lung cancer (NSCLC) genetic profiles 7. Based on molecular studies, the expression of some genes in LCNEC (STK11, KEAP1) was similar to that in NSCLC; therefore, LCNEC was more likely to be a hybrid subtype of SCLC and NSCLC 8. Controversially, the characteristics of early stage LCNEC appear to be consistent with NSCLC when analyzed from clinical features, whereas in stage IV LCNEC the metastatic pattern resembles that of SCLC 9. Advanced SCLC and advanced LCNEC are often indicative of tumors with organ metastases, their prognosis is extremely poor and their treatment has always been challenging. The median OS (mOS) for stage IV SCLC was reported to be 7.0 months, with a 3-year survival rate of only 7.2% 10, and the mOS for stage IV LCNEC was 4.0 (3.5-4.6) months 9, which is lower than that for other types of lung cancer. In addition, the treatments for advanced LCENC have been contradictory. Some studies suggest that advanced LCNEC should be treated with the SCLC-type modality because the SCLC-type modality shows better OS and progression-free survival (PFS) 11, 12, yet others oppose it 13.

Advanced LCNEC is a rare and aggressive type of cancer. Whether patients with stage IV LCNEC should be treated as NSCLC or SCLC remains controversial 14. The Surveillance, Epidemiology, and End Results (SEER) database is unique in the number of cases, especially for LCNEC patients. In this study, we obtained stage IV SCLC and IV NSCLC data from SEER database and performed 1:1 propensity score matching (PSM) analysis to compare the clinical characteristics, prognostic factors and treatment modalities of advanced LCNEC and SCLC.

## Materials and Methods

### Data collection

SEER is a United States cancer patient-based database that collects data on approximately 30% of all cancer patients with the goal of reducing the burden of cancer (https://seer.cancer.gov/). LCNEC and SCLC data were downloaded from Incident SEER Research Plus Data, 17 Registries, Nov 2021 Sub (2000-2015). Inclusion criteria: (1) all subjects were diagnosed in 2010-2019; (2) age > 18; (3) primary site (ICD-O-3 /WHO 2008): lung and bronchus; (4) histology code (ICD-O-3 Hist/behav): 8013/3, 8002/3, 8041/3, 8042/3, 8043/3 and 8044/3. (5) The tumor stage was IV. Exclusion criteria: (1) Follow-up data unknown and missing; (2) Incomplete clinical data and other relevant information.

**Figure 1:**
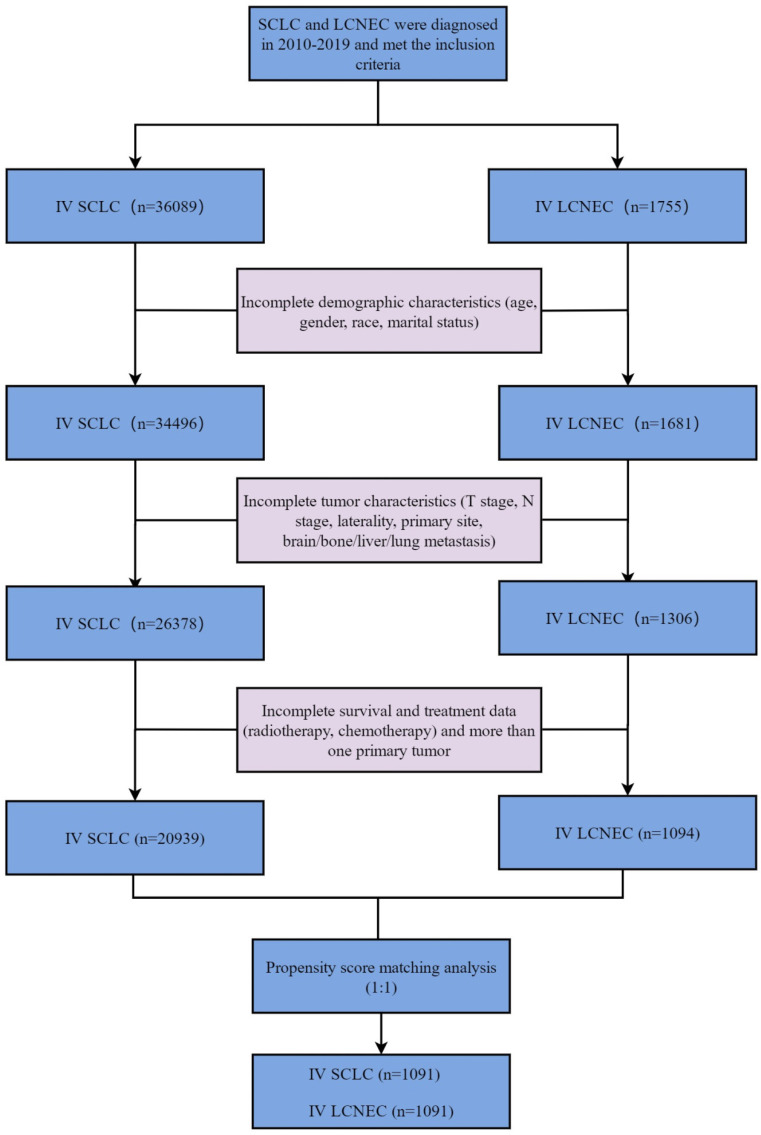
Flow chart for screening patients. LCNEC: large cell neuroendocrine carcinoma; SCLC: small cell lung cancer.

The variables collected included demographic characteristics of patients: age, gender, race, marital status. Tumor characteristics: laterality, T stage, N stage, brain metastasis, bone metastasis, liver metastasis, lung metastasis, primary site. Treatment: Radiation, chemotherapy. Survival data: Survival months, overall survival (OS) and cancer-specific survival (CSS). To facilitate statistical analysis, we reclassified some variables: age (≥ 65,< 65), marital status (married, unmarried), T stage (T0,T1,T2,T3,T4), N stage (N0,N1,N2,N3), laterality (left, right, others), radiotherapy (Yes, No), primary site (main bronchus, upper lobe, middle lobe, lower lobe, others). OS and CSS were the primary endpoints in this study. Patients diagnosed in 2016-2017 were reclassified to T stage and N stage according to the "2016 SEER Manual Section V: Stage of Disease at Diagnosis" document. Definition of Treatment options: (1) Radiotherapy: Yes: patients were treated with radiotherapy as first course of treatment. No: patients were not treated with radiotherapy as first course of treatment. (2) Chemotherapy: Yes: patients were treated with chemotherapy. No: patients were not treated with chemotherapy. (3) Chemoradiotherapy: Yes: patients were both treated with radiotherapy and chemotherapy. No: patients were treated with radiotherapy/chemotherapy alone or were given neither chemotherapy nor radiotherapy. The flow chart of patient screening is shown in Figure 1.

### Propensity score matching

**Figure 2:**
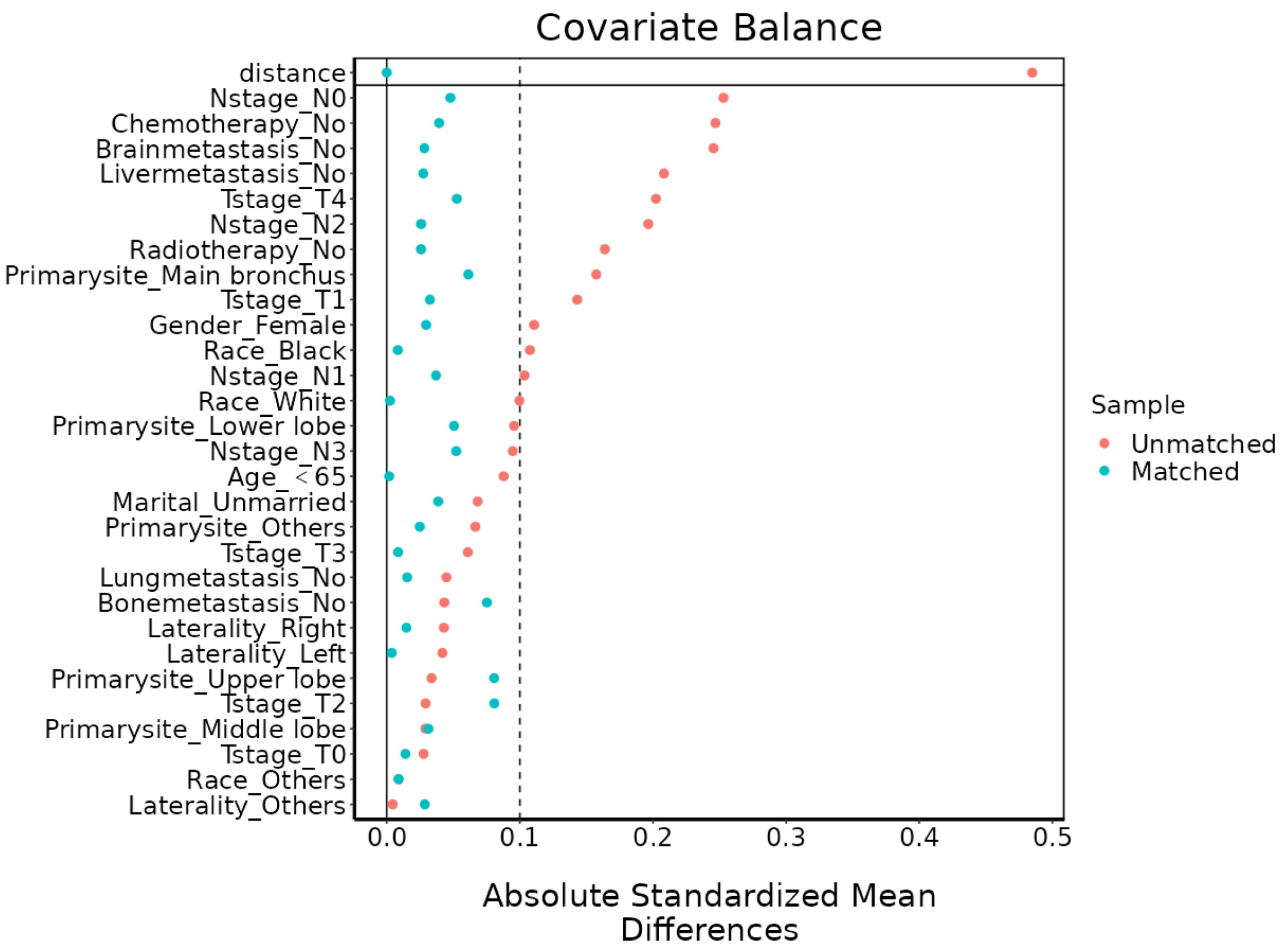
Standardized mean differences before and after PSM. PSM: propensity score matching.

To reduce the effect of selection bias, PSM was applied to SCLC and LCNEC groups in this study. The matching ratio for stage IV SCLC and LCNEC groups was 1:1 and the caliper value was set to 0.03 through the “nearest” method (Figure 2). The variables used for matching were as follows: age, gender, race, marital status, T stage, N stage, laterality, primary site, brain metastasis, bone metastasis, liver metastasis, lung metastasis, radiotherapy, chemotherapy.

### Statistical Methods

All statistical analyses were performed with SPSS 23.0(SPSS Inc., Chicago, IL, USA) and R version 4.2.1. P-value < 0.05 was considered statistically significant. The "MatchIt" package of R was used to perform PSM analyses. Kaplan-Meier (KM) analysis was used to compare the prognosis of different treatment modalities. Pearson's χ2 test was utilized to compare the baseline characteristics of the stage IV SCLC and LCNEC groups. Univariable and multivariate Cox proportional hazard models were used to identify risk factors for OS and CSS in the stage IV LCNEC and SCLC groups.

## Results

### Basic characteristics of patients

In this study, a total of 22033 patients were diagnosed from 2010 to 2019, including stage IV SCLC (n=20939) and stage IV LCNEC (n=1094) groups (Table 1). The basic characteristics of the patients are shown in Table 1. Compared with the stage IV SCLC group, age, gender, race, marital status, T stage, N stage, primary site, brain metastasis, liver metastasis, radiotherapy and chemotherapy were significantly different in stage IV LCNEC group before PSM (p < 0.05). T4 (42.4%), N2 (53.7%) and N3 (27.1%) were common in IV SCLC patients. Brain metastases (38.4% vs 26.5%) were more common and liver metastases (32.3% vs 42.0%) were less common in IV LCNEC than in SCLC. More IV LCNEC patients chose radiotherapy (54.6% vs 46.4%) and fewer patients chose chemotherapy (68.1% vs 79.6%) than IV SCLC patients.

The stage IV SCLC group (n=1091) and stage IV LCNEC group (n=1091) were selected for further analysis after 1:1 PSM, and the baseline features were well-balanced between the IV SCLC and IV LCNEC groups (Table 2).

### KM analysis for IV LCNEC and IV SCLC

**Figure 3:**
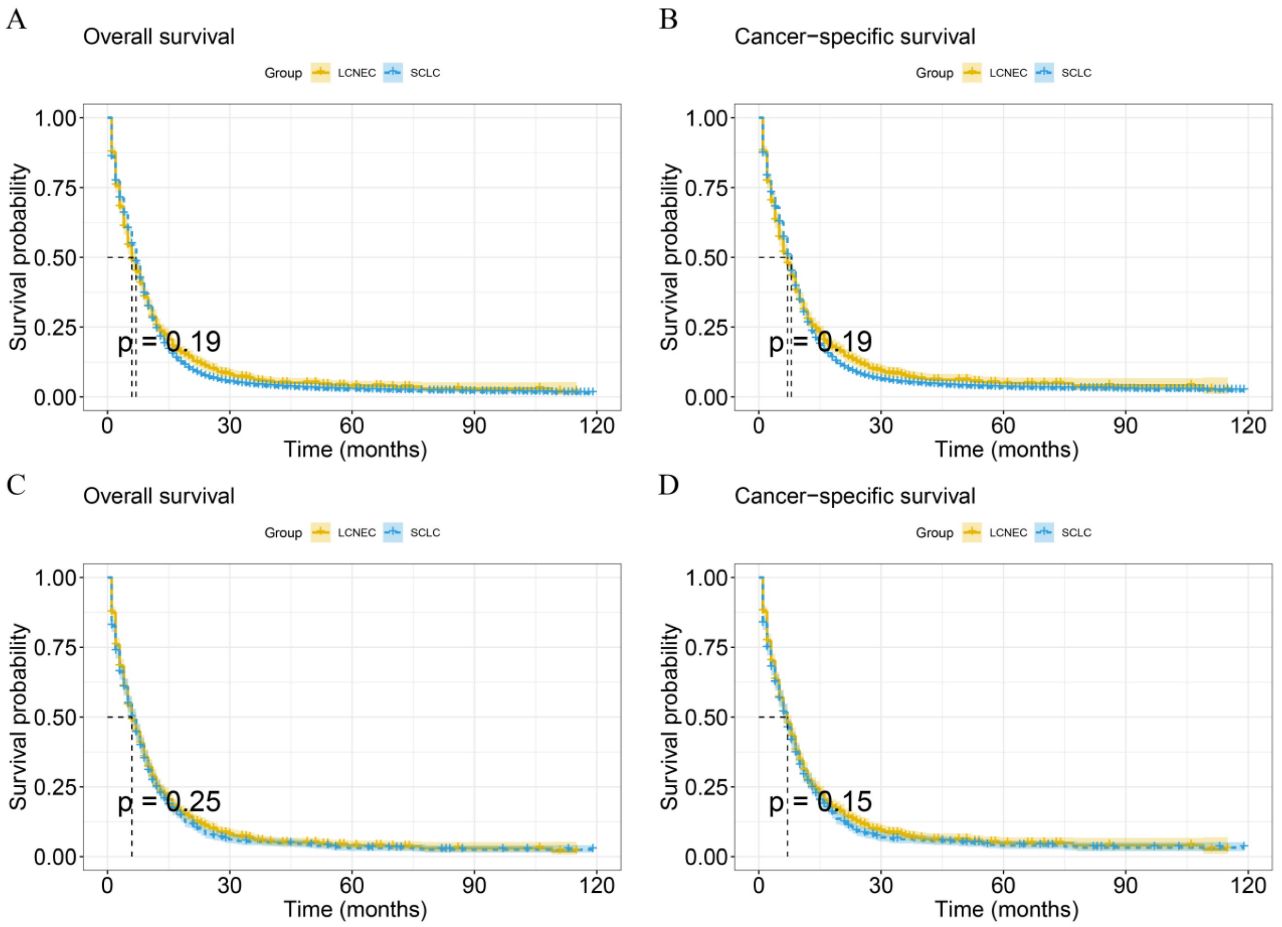
KM curves in IV LCNEC and IV SCLC patients before and after PSM. A: The KM curve of OS before PSM (p=0.19). B: The KM curve of CSS before PSM (p=0.19). C: The KM curve of OS after PSM (p=0.25). D: The KM curve of CSS after PSM (p=0.15). LCNEC: large cell neuroendocrine carcinoma; SCLC: small cell lung cancer; OS: overall survival; CSS: cancer-specific survival; PSM: propensity score matching; KM: Kaplan-Meier.

KM analysis was used to compare OS or CSS in the stage IV LCNEC and stage IV SCLC groups (Figure 3). The mOS of IV LCNEC was 6.0(95%CL: 5.44-6.56 months), IV SCLC was 7.0 (95%CL: 6.89-7.11 months); median CSS (mCSS) of IV LCNEC was 7.0 (95%CL: 6.34-7.66 months), IV SCLC was 8.0 (95%CL: 7.89-8.11 months). There was no statistically significant difference in OS (p = 0.19) or CSS (p = 0.19) between stage IV LCNEC and stage IV SCLC groups before PSM. After PSM, the mOS of IV LCNEC was 6.0 (95%CL: 5.44-6.56 months), IV SCLC was 6.0 (95%CL: 5.45-6.55 months); the mCSS of IV LCNEC was 7.0 (95%CL: 6.34-7.66 months), IV SCLC was 7.0 (95%CL: 6.40-7.61 months). There was no statistically significant difference in OS (p = 0.25) and CSS (p = 0.15) between the stage IV LCNEC and stage IV SCLC groups after PSM. The 1-year, 2-year and 3-year survival rates are shown in Table 3.

### Univariable Cox analysis for IV LCNEC and IV SCLC after PSM

Univariate Cox analysis was performed with OS and CSS in stage IV LCNEC and stage IV SCLC patients after PSM. The results of univariate Cox analysis showed that age, N stage, marital status, primary site, radiotherapy, chemotherapy, brain metastasis, liver metastasis was significantly associated with OS in stage IV SCLC and stage IV LCNEC patients (Table 4); Besides, T stage was significantly associated with OS in stage IV SCLC patients, bone metastasis was significantly associated with OS in stage IV LCNEC patients. Age, N stage, primary site, brain metastasis, liver metastasis, radiotherapy, and chemotherapy were correlated with CSS of IV LCNEC and IV SCLC after PSM (Table 5). Besides, T stage was correlated with CSS of SCLC; bone metastasis was correlated with CSS of LCNEC.

### Multivariate Cox analysis for IV LCNEC and IV SCLC after PSM

**Figure 4:**
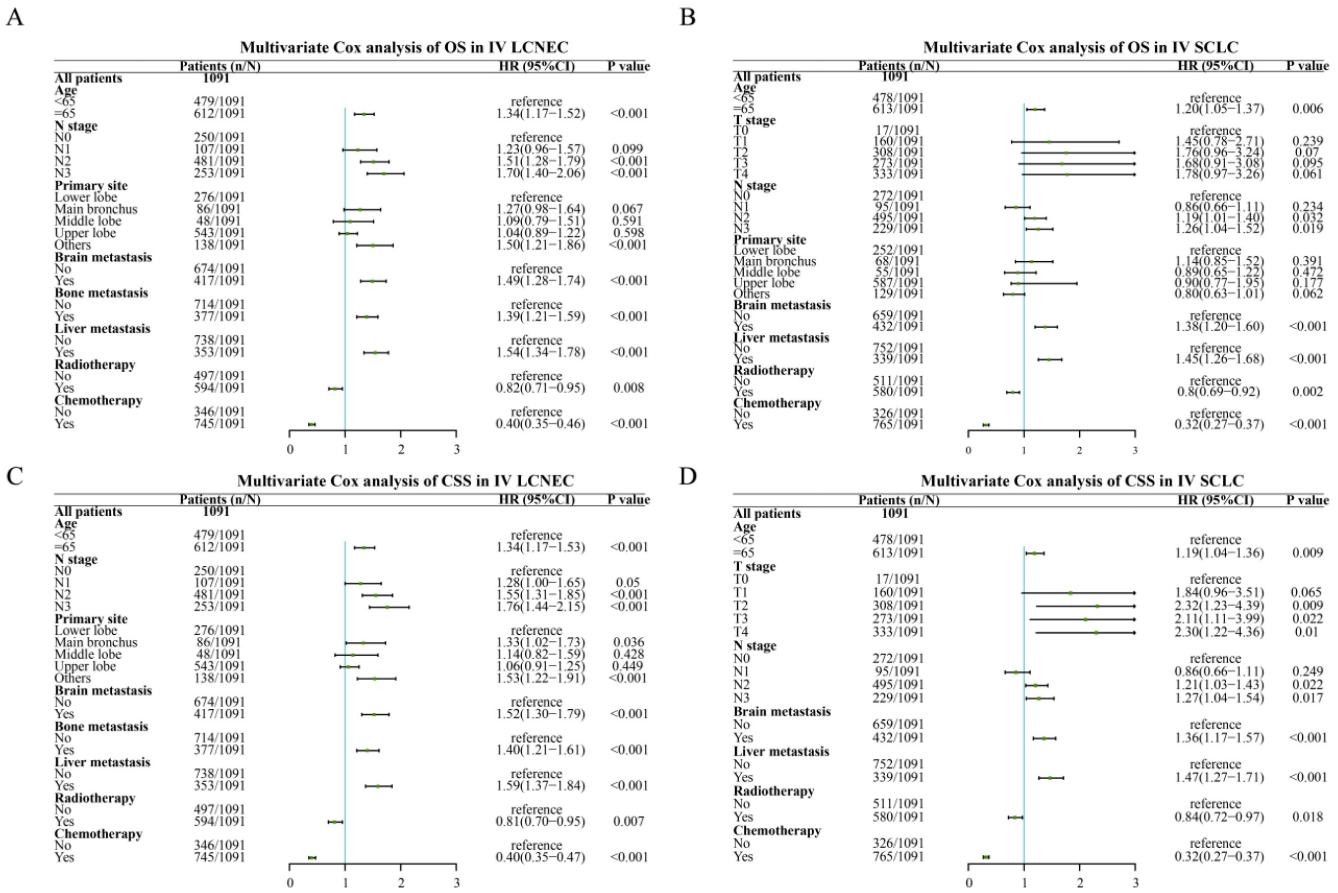
The multivariate Cox analysis of IV LCNEC and IV SCLC after PSM. A: The multivariate Cox analysis of OS in IV LCNEC patients; B: The multivariate Cox analysis of OS in IV SCLC patients; C: The multivariate Cox analysis of CSS in IV LCNEC patients; D: The multivariate Cox analysis of CSS in IV SCLC patients. LCNEC: large cell neuroendocrine carcinoma; SCLC: small cell lung cancer; OS: overall survival; CSS: cancer-specific survival; PSM: propensity score matching.

In the multivariate Cox analysis with stage IV LCNEC and IV SCLC patients, age, N stage, brain metastasis, liver metastasis were independent risk factors for OS while radiotherapy, chemotherapy were common independent protective factors for OS in stage IV SCLC and stage IV LCNEC patients (Figure 4). Besides, liver metastasis was only an independent risk factor for IV LCNEC patients. In addition, age, N stage, primary site, brain metastasis, liver metastasis were common independent risk/protective factors for CSS in IV SCLC and IV LCNEC patients. T stage was an independent risk factor for CSS in SCLC and bone metastasis was an independent risk factor for CSS in LCNEC.

### Prognosis of each treatment modality in IV LCNEC patients

**Figure 5:**
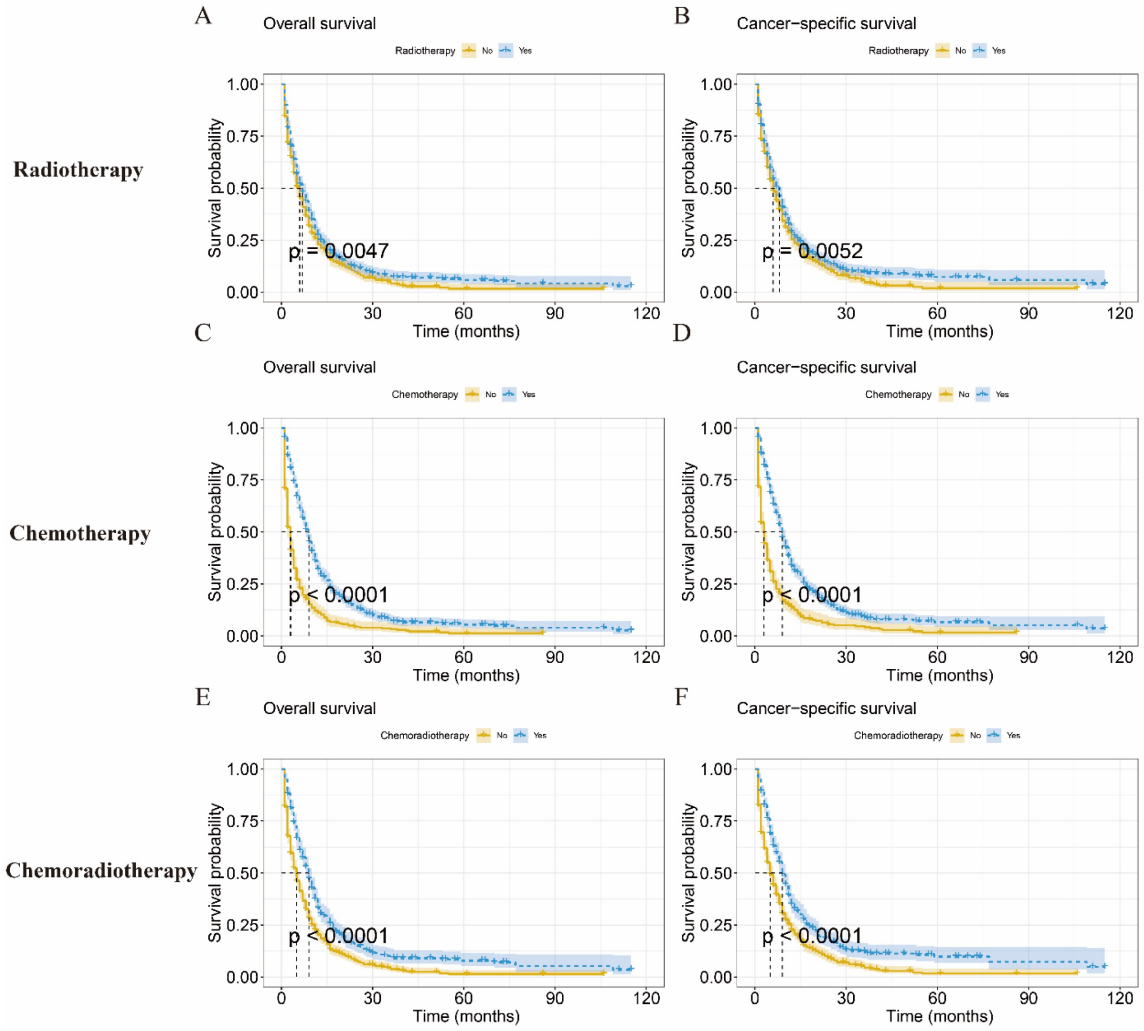
KM curves of IV LCNEC patients with each treatment modality. Kaplan-Meier curve of OS in radiotherapy (A), chemotherapy (C), and chemoradiotherapy (E) for IV LCNEC patients. Kaplan-Meier curve of CSS in radiotherapy (B), chemotherapy (D), and chemoradiotherapy (F) for IV LCNEC patients. OS: overall survival; CSS: cancer-specific survival; LCNEC: large cell neuroendocrine carcinoma; KM: Kaplan-Meier.

To assess the prognostic impact of each treatment modality on patients with IV LCNEC, we compared treatment outcomes with radiotherapy, chemotherapy, and chemoradiotherapy, respectively. The mOS of radiotherapy was 7.0 months (95%CI: 6.08-7.91months) (Figure 5A), chemotherapy was 9.0 months (95%CI: 8.31-9.69months) (Figure 5B), chemoradiotherapy was 9.0 months (95%CI: 8.01-9.98 months) (Figure 5C).The KM analysis showed that radiotherapy, chemotherapy and chemoradiotherapy could improve the survival probability of IV LCNEC patients (p < 0.05). The mCSS of radiotherapy, chemotherapy and chemoradiotherapy were 8.0 months (95%CI: 7.13-8.88 months), 9.0 months (95%CI: 8.32-9.69 months) and 9.0 months (95%CI: 8.09-9.92 months) respectively (Figure 5D-F).

### Evaluation of different treatment modalities of IV SCLC and IV LCNEC

To identify the effect of treatment modalities on OS and CSS for stage IV SCLC and stage IV LCNEC, patients were divided into four groups according to treatment modalities before PSM: Control: patients were not treated with radiotherapy or chemotherapy since being diagnosed. Radiotherapy: patients were treated with radiotherapy alone. Chemotherapy: patients were treated with chemotherapy alone. Chemoradiotherapy: patients were both treated with radiotherapy and chemotherapy. The baseline characteristics of IV SCLC and IV LCNEC were shown in Table S1 and Table S2.

**Figure 6:**
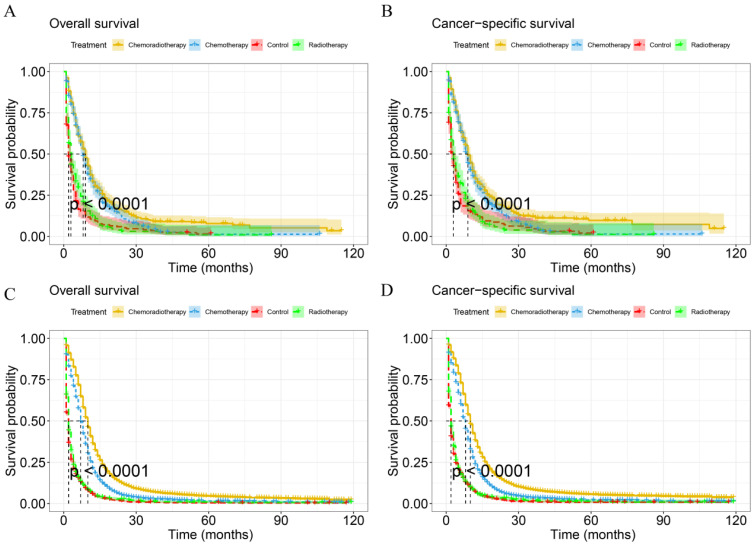
KM curves for IV LCNEC and IV SCLC in different treatment modalities. KM curves of OS (A) and CSS (B) for IV LCNEC in different treatment modalities. KM curves of OS(C) and CSS (D) for IV SCLC in different treatment modalities. LCNEC: large cell neuroendocrine carcinoma; SCLC: small cell lung cancer; OS: overall survival; CSS: cancer-specific survival; KM: Kaplan-Meier.

KM analysis was used to compare the difference in survival probability between patients with different treatment modalities (Figure 6). The mOS and mCSS of chemoradiotherapy group were 10.0 months (95%CI: 9.81-10.19 months) and 10.0 months (9.81-10.19 months) respectively, with a better survival probability than other groups in IV SCLC (p < 0.001). Besides, chemotherapy and radiotherapy alone had better OS and CSS than the control group in IV SCLC (p<0.05). For IV LCNEC patients, the mOS and mCSS in the chemoradiotherapy group were 9.0 months (95%CI: 8.02-9.99 months) and 9.0 months (95%CI: 8.09-9.92 months) respectively, which had a higher probability of survival than the other treatment groups (p < 0.001). In addition, the OS and CSS of the chemotherapy group were better than those of the control group (p < 0.001). However, the OS and CSS of radiotherapy alone were not significantly different compared to the control group in IV LCNEC patients (p = 0.228, p = 0.391).

## Discussion

Similar histological features of LCNEC and SCLC were demonstrated in some studies 15, and further exploration of the differences in clinical features, prognostic factors, and treatment modalities between LCNEC and SCLC is warranted, especially for advanced LCNEC patients. Our study confirmed significant differences in the clinical characteristics of advanced LCNEC and SCLC, as reflected by demographic characteristics and tumor characteristics. Furthermore, after PSM, there was no significant difference in OS and CSS between IV LCNEC and IV SCLC, and their risk/protective factors were broadly similar based on the results of univariable and multivariable proportional hazards regression analysis, except for T stage and bone metastasis. In terms of treatment modality, chemoradiotherapy was the optimal treatment modality for advanced LCNEC and advanced SCLC patients, with better OS and CSS than other treatment modalities. However, radiotherapy alone could benefit advanced SCLC patients but did not seem to benefit advanced LCNEC patients.

Varlotto conducted a large retrospective study that included patients with LCNEC, SCLC and NSCLC from 2001-2007 and they concluded that the clinical features of LCNEC were more inclined to NSCLC than to SCLC 13. Wang identified significant differences in both demographic and clinical characteristics of LCNEC from SCLC, except for marital status 16. To our knowledge, our study was the first to confirm that there are significant differences in demographics and treatment modalities between LCNEC and SCLC at an advanced stage of the disease. However, Isaka suggested that the clinical characteristics of LCNEC and SCLC were similar 17, his study included only 10 patients with LCNEC, which may be biased by the small sample size. Derks found a similar pattern of organ metastases in advanced LCNEC as in SCLC, but liver metastases were less common in SCLC 9. Our study found that brain metastases were common in advanced LCNEC patients compared with advanced SCLC patients, although the brain was reported to be the most common organ of metastasis in SCLC 18, which suggests that brain metastases may be more common in advanced LCNEC patients. In addition, Derks' study reported a higher probability of brain metastasis in advanced LCNEC/SCLC than in NSCLC 9. Radiotherapy was more common in advanced LCNEC than SCLC, while previous studies have shown that SCLC appears to be more sensitive to radiotherapy 19, 20. This may because LCENC with organ metastases is often recommended as the primary treatment with postsurgical radiotherapy 21, while chemotherapy combined with immunotherapy is preferred for advanced SCLC 22, which also increases the proportion of patients with IV LCNEC treated with radiotherapy. In the future, with the improvement of pathological detection techniques, LCNEC patients continue to be identified, and the differences with SCLC may change as the number of patient increases.

SCLC is considered to have the worst prognosis and highest malignancy of all lung cancer tissue types, especially in the extensive stage 23; nevertheless, our study suggested that advanced LCNEC may also be highly malignant. The prognosis of SCLC and LCNEC remains controversial, Tomonari found no significant difference in OS and PFS between SCLC and LCNEC patients after surgery 24; however, Varlotto found that the 1-, 2-, and 4-year OS probabilities of LCNEC (76%, 56%, 41%) were significantly higher than those of SCLC (69%, 49%, 32%) in patients undergoing surgery without radiotherapy, which was similar to the prognosis of NSCLC 13. Isaka also reported a better prognosis for stage IA LCNEC than for SCLC patients with small-sized tumors 17. Nevertheless, few studies have compared the prognosis of LCNEC and SCLC with different stages. Derks reported that LCNEC presented a better OS than SCLC in the early-stage disease but presented a similar OS to SCLC in the advanced stage 9. Our study favors Derks' finding that OS and CSS were not significantly different between patients with advanced LCENC and SCLC, and similar results were reported after PSM analysis. Although the prognostic differences between studies on LCNEC and SCLC were inconsistent, our study and most studies confirmed that the prognosis of LCNEC and SCLC is similar, especially in the advanced stages of the disease.

We confirmed that OS and CSS prognostic factors for IV LCNEC and IV SCLC were similar after PSM, ranging from demographic characteristics to tumor characteristics. Risk factors for SCLC have been widely reported in other studies 18, 25. The prognostic factors for LCNEC have received increasing attention in recent years, and age, gender, insurance, marital status, and tumor size have been confirmed as risk factors for LCNEC 26-28. Unlike other studies, gender, race, and marriage were not reported to correlate with the prognosis of IV LCNEC, nor was IV SCLC in this study. In addition, chemotherapy and radiotherapy prolonged OS and CSS and improved prognosis in IV LCNEC and IV SCLC, which is consistent with IV NSCLC 9.

At present, the standard treatment of advanced LCNEC remains controversial, and also underappreciated due to its low morbidity 16. Varlotto compared the characteristics of patients with LCNEC, SCLC, and NSCLC and confirmed that the characteristics and prognosis of LCNEC were more similar to those of NSCLC; therefore, he concluded that treatment of LCNEC should continue with NSCLC regimens 13. Unfortunately, he did not control for sample selection bias, which may eventually lead to biased results; secondly, he did not compare the characteristics and prognosis of advanced LCNEC. Sun conducted a study, in which patients with LCNEC were treated with SCLC regimens and NSCLC regimens, and the results showed a better prognosis in the SCLC regimens group; thus, he concluded that LCNEC should be treated with the SCLC regimens 12. Derks suggested that early stage LCNEC treatment strategy should refer to the treatment of NSCLC regimens, while advanced stage LCNEC should be treated with SCLC regimens 9. Previous studies have explored the treatment of stage IV LCNEC, but due to sample size limitations, there are still no consistent conclusions 2. Our results confirmed that advanced LCNEC and SCLC benefit to a similar extent in each treatment modality based on a large sample of IV LCNEC patients, suggesting that the treatment of stage IV LCNEC patients might favor SCLC regimens.

There are still several limitations in our study. First, some patient information such as smoking, specific chemotherapy and radiation regimens, immunotherapy and targeted therapies were not provided in the SEER database, which may have had an impact on our results. Second, the information bias introduced by retrospective studies may cause errors in our results. Third, we failed to present information on stage IV LCNEC patients from our own database due to the limitations of diagnostic techniques and the low morbidity and high mortality of LCNEC. More prospective studies are needed to further explore the similarity between SCLC and LCNEC in the future.

## Conclusion

The clinical features of advanced LCNEC differ from those of advanced SCLC, while the survival outcomes and treatment modalities are similar. In summary, advanced LCNEC is similar to advanced SCLC, and advanced LCNEC could be treated with advanced SCLC regimens, which provide new evidence for the treatment of advanced LCNEC patients.

## Supplementary Material

Supplementary tables.

## Figures and Tables

**Table 1:** Patient characteristics before PSM

Variable	SCLC	LCNEC	p-value
All participants	20939 (100.0%)	1094 (100.0%)	
Age(years)			0.005
≥65	12664 (60.5%)	614 (56.1%)	
<65	8275 (39.5%)	480 (43.9%)	
Gender			<0.001
Male	10699 (51.1%)	619 (56.6%)	
Female	10240 (48.9%)	475 (43.4%)	
Race			<0.001
Black	1860 (8.9%)	136 (12.4%)	
White	18180 (86.8%)	909 (83.1%)	
Others	899 (4.3%)	49 (4.5%)	
Marital status			0.031
Married	10772 (51.4%)	600 (54.8%)	
Unmarried	10167 (48.6%)	494 (45.2%)	
T stage			<0.001
T0	288 (1.4%)	19 (1.7%)	
T1	2268 (10.8%)	176 (16.1%)	
T2	4904 (23.4%)	270 (24.7%)	
T3	4599 (22%)	269 (24.6%)	
T4	8880 (42.4%)	360 (32.9%)	
N stage			<0.001
N0	2609 (12.5%)	253 (23.1%)	
N1	1404 (6.7%)	107 (9.8%)	
N2	11248 (53.7%)	481 (44%)	
N3	5678 (27.1%)	253 (23.1%)	
Laterality			0.381
Left	8677 (41.4%)	431 (39.4%)	
Right	11440 (54.6%)	621 (56.8%)	
Others	822 (3.9%)	42 (3.8%)	
Primary site			<0.001
Main bronchus	2533 (12.1%)	86 (7.9%)	
Upper lobe	10059 (48%)	544 (49.7%)	
Middle lobe	794 (3.8%)	48 (4.4%)	
Lower lobe	4449 (21.2%)	278 (25.4%)	
Others	3104 (14.8%)	138 (12.6%)	
Brain Metastasis			<0.001
Yes	5539 (26.5%)	420 (38.4%)	
No	15400 (73.5%)	674 (61.6%)	
Bone Metastasis			0.179
Yes	7646 (36.5%)	377 (34.5%)	
No	13293 (63.5%)	717 (65.5%)	
Liver Metastasis			<0.001
Yes	8795 (42%)	353 (32.3%)	
No	12144 (58%)	741 (67.7%)	
Lung Metastasis			0.147
Yes	4335 (20.7%)	247 (22.6%)	
No	16604 (79.3%)	847 (77.4%)	
Radiotherapy			<0.001
Yes	9718 (46.4%)	597 (54.6%)	
No	11221 (53.6%)	497 (45.4%)	
Chemotherapy			<0.001
Yes	16669 (79.6%)	745 (68.1%)	
No	4270 (20.4%)	349 (31.9%)	

Abbreviations: PSM: propensity score matching, SCLC: small cell lung cancer, LCNEC: large cell neuroendocrine carcinoma.

**Table 2:** Patient characteristics after PSM

Variable	SCLC	LCNEC	p-value
All participants	1091(100.0%)	1091(100.0%)	
Age(years)			1.000
≥65	613 (56.2%)	612 (56.1%)	
<65	478 (43.8%)	479 (43.9%)	
Gender			0.518
Male	600 (55%)	616 (56.5%)	
Female	491 (45%)	475 (43.5%)	
Race			0.963
Black	138 (12.6%	135 (12.4%)	
White	907 (83.1%))	908 (83.2%)	
Others	46 (4.2%)	48 (4.4%)	
Marital status			0.390
Married	577 (52.9%)	598 (54.8%)	
Unmarried	514 (47.1%)	493 (45.2%)	
T stage			0.380
T0	17 (1.6%)	19 (1.7%)	
T1	160 (14.7%)	173 (15.9%)	
T2	308 (28.2%)	270 (24.7%)	
T3	273 (25%)	269 (24.7%)	
T4	333 (30.5%)	360 (33%)	
N stage			0.386
N0	272 (24.9%)	250 (22.9%)	
N1	95 (8.7%)	107 (9.8%)	
N2	495 (45.4%)	481 (44.1%)	
N3	229 (21%)	253 (23.2%)	
Laterality			0.772
Left	428 (39.2%)	430 (39.4%)	
Right	627 (57.5%)	619 (56.7%)	
Others	36 (3.3%)	42 (3.8%)	
Primary site			0.224
Main bronchus	68 (6.2%)	86 (7.9%)	
Upper lobe	587 (53.8%)	543 (49.8%)	
Middle lobe	55 (5%)	48 (4.4%)	
Lower lobe	252 (23.1%)	276 (25.3%)	
Others	129 (11.8%)	138 (12.6%)	
Brain Metastasis			0.539
Yes	432 (39.6%)	417 (38.2%)	
No	659 (60.4%)	674 (61.8%)	
Bone Metastasis			0.083
Yes	338 (31%)	377 (34.6%)	
No	753 (69%)	714 (65.4%)	
Liver Metastasis			0.550
Yes	339 (31.1%)	353 (32.4%)	
No	752 (68.9%)	738 (67.6%)	
Lung Metastasis			0.758
Yes	240 (22%)	247 (22.6%)	
No	851 (78%)	844 (77.4%)	
Radiotherapy			0.577
Yes	580 (53.2%)	594 (54.4%)	
No	511 (46.8%)	497 (45.6%)	
Chemotherapy			0.378
Yes	765 (70.1%)	745 (68.3%)	
No	326 (29.9%)	346 (31.7%)	

Abbreviations: PSM: propensity score matching, SCLC: small cell lung cancer, LCNEC: large cell neuroendocrine carcinoma.

**Table 3:** The survival probability of OS and CSS in IV LCNEC and IV SCLC

Before PSM	OS		CSS	
	LCNEC	SCLC	LCNEC	SCLC
1-year survival probability	25.29%	24.15%	27.46%	26.30%
2-year survival probability	11.07%	7.34%	12.58%	8.46%
3-year survival probability	6.12%	4.21%	7.47%	5.04%
After PSM				
1-year survival probability	25.22%	24.64%	27.39%	26.82%
2-year survival probability	11.01%	8.12%	12.52%	9.09%
3-year survival probability	6.09%	5.35%	7.44%	6.25%

Abbreviations: OS: overall survival, CSS: cancer-specific survival, PSM: propensity score matching, SCLC: small cell lung cancer, LCNEC: large cell neuroendocrine carcinoma.

**Table 4:** Univariable Cox analysis of OS in IV SCLC and IV LCNEC after PSM

Variable	SCLC		LCNEC	
	HR (95% CI)	p-value	HR (95% CI)	p-value
Age				
<65				
≥65	1.30 (1.15-1.48)	<0.001	1.36 (1.20-1.55)	<0.001
Gender				
Female				
Male	1.10 (0.97-1.24)	0.146	1.10 (0.97-1.25)	0.139
Race				
Black				
White	1.10 (0.91-1.33)	0.311	1.16 (0.96-1.41)	0.129
Others	1.40 (0.99-1.99)	0.060	0.93 (0.65-1.32)	0.679
Marital status				
Unmarried				
Married	0.91 (0.80-1.03)	0.123	0.92 (0.81-1.04)	0.179
T stage				
T0				
T1	1.79 (0.99-3.22)	0.054	1.15 (0.69-1.90)	0.595
T2	1.95 (1.09-3.47)	0.024	1.27 (0.78-2.08)	0.341
T3	1.88 (1.05-3.36)	0.033	1.53 (0.94-2.51)	0.090
T4	1.76 (0.99-3.14)	0.054	1.54 (0.94-2.51)	0.086
N stage				
N0				
N1	0.87 (0.68-1.12)	0.296	1.18 (0.93-1.50)	0.176
N2	1.19 (1.02-1.39)	0.029	1.38 (1.17-1.62)	<0.001
N3	1.13 (0.94-1.36)	0.193	1.40 (1.16-1.69)	<0.001
Laterality				
Left				
Right	0.99 (0.87-1.12)	0.833	1.05 (0.92-1.20)	0.443
Others	0.73 (0.50-1.07)	0.103	1.05 (0.76-1.45)	0.779
Primary site				
Lower lobe				
Main bronchus	0.96 (0.72-1.27)	0.770	1.11 (0.86-1.43)	0.434
Middle lobe	0.90 (0.66-1.22)	0.485	0.98 (0.71-1.35)	0.888
Upper lobe	0.84 (0.72-0.99)	0.032	0.93 (0.80-1.09)	0.379
Others	0.87 (0.70-1.09)	0.238	1.29 (1.05-1.60)	0.018
Brain Metastasis				
No				
Yes	1.16 (1.03-1.32)	0.019	1.17 (1.03-1.33)	0.017
Bone Metastasis				
No				
Yes	1.12 (0.97-1.28)	0.112	1.39 (1.22-1.59)	<0.001
Liver Metastasis				
No				
Yes	1.44 (1.26-1.65)	<0.001	1.47 (1.28-1.68)	<0.001
Lung Metastasis				
No				
Yes	1.02 (0.88-1.19)	0.779	1.09 (0.94-1.27)	0.235
Radiotherapy				
No				
Yes	0.70 (0.61-0.79)	<0.001	0.84 (0.74-0.95)	0.005
Chemotherapy				
No				
Yes	0.32 (0.28-0.37)	<0.001	0.43 (0.37-0.49)	<0.001

Abbreviations: OS: overall survival, PSM: propensity score matching, SCLC: small cell lung cancer, LCNEC: large cell neuroendocrine carcinoma.

**Table 5:** Univariable Cox analysis of CSS in IV SCLC and IV LCNEC after PSM

Variable	SCLC		LCNEC	
	HR (95% CI)	p-value	HR (95% CI)	p-value
Age				
<65				
≥65	1.28 (1.13-1.46)	<0.001	1.36 (1.19-1.55)	<0.001
Gender				
Female				
Male	1.11 (0.97-1.26)	0.121	1.11 (0.98-1.27)	0.113
Race				
Black				
White	1.08 (0.89-1.31)	0.430	1.19 (0.98-1.46)	0.085
Others	1.34 (0.93-1.92)	0.116	0.97 (0.67-1.39)	0.859
Marital status				
Unmarried				
Married	0.92 (0.81-1.05)	0.221	0.93 (0.82-1.06)	0.273
T stage				
T0				
T1	1.99 (1.05-3.79)	0.036	1.12 (0.66-1.88)	0.679
T2	2.23 (1.19-4.20)	0.013	1.24 (0.74-2.06)	0.414
T3	2.10 (1.12-3.96)	0.022	1.55 (0.93-2.58)	0.090
T4	2.03 (1.08-3.82)	0.028	1.52 (0.92-2.52)	0.104
N stage				
N0				
N1	0.87 (0.67-1.13)	0.285	1.24 (0.97-1.58)	0.091
N2	1.20 (1.03-1.41)	0.023	1.42 (1.20-1.68)	<0.001
N3	1.15 (0.95-1.39)	0.155	1.45 (1.20-1.77)	<0.001
Laterality				
Left				
Right	0.99 (0.86-1.12)	0.832	1.04 (0.91-1.19)	0.558
Others	0.66 (0.44-0.99)	0.045	1.00 (0.71-1.41)	0.991
Primary site				
Lower lobe				
Main bronchus	0.98 (0.74-1.31)	0.911	1.15 (0.89-1.50)	0.283
Middle lobe	0.92 (0.67-1.26)	0.588	1.01 (0.73-1.41)	0.936
Upper lobe	0.86 (0.73-1.01)	0.064	0.95 (0.81-1.11)	0.527
Others	0.85 (0.67-1.07)	0.162	1.31 (1.05-1.64)	0.015
Brain Metastasis				
No				
Yes	1.16 (1.02-1.32)	0.024	1.18 (1.03-1.35)	0.015
Bone Metastasis				
No				
Yes	1.14 (0.99-1.31)	0.061	1.40 (1.23-1.61)	<0.001
Liver Metastasis				
No				
Yes	1.45 (1.26-1.66)	<0.001	1.51 (1.32-1.74)	<0.001
Lung Metastasis				
No				
Yes	1.02 (0.88-1.19)	0.794	1.11 (0.95-1.29)	0.188
Radiotherapy				
No				
Yes	0.71(0.63-0.81)	<0.001	0.83 (0.73-0.95)	0.006
Chemotherapy				
No				
Yes	0.32 (0.27-0.37)	<0.001	0.43 (0.38-0.50)	<0.001

Abbreviations: CSS: cancer-specific survival, PSM: propensity score matching, SCLC: small cell lung cancer, LCNEC: large cell neuroendocrine carcinoma.
