# Supplementary material for: Differences between Advanced Large Cell Neuroendocrine Carcinoma and Advanced Small Cell Lung Cancer: A Propensity Score Matching Analysis
Source: J Cancer. 2023 May 21;14(9):1541–52. doi: 10.7150/jca.84600 (PMC10266246; doi:10.7150/jca.84600)
Supplement: Supplementary file 1 — Supplementary tables. [file jcav14p1541s1.pdf]

Supplementary files

Table S1. The baseline characteristics of different treatment modalities in IV LCNEC

Variable	Control (N=179)	Radiotherapy (N=170)	Chemotherapy (N=318)	Chemoradiotherapy (N=427)	p-value
Age					<0.001
＜65	129 (72.1%)	104 (61.2%)	177 (55.7%)	204 (47.8%)	
≥65	50 (27.9%)	66 (38.8%)	141 (44.3%)	223 (52.2%)	
Gender					0.389
Male	92 (51.4%)	94 (55.3%)	188 (59.1%)	245 (57.4%)	
Female	87 (48.6%)	76 (44.7%)	130 (40.9%)	182 (42.6%)	
Race					0.709
Black	23 (12.8%)	20 (11.8%)	32 (10.1%)	61 (14.3%)	
White	150 (83.8%)	142 (83.5%)	270 (84.9%)	347 (81.3%)	
Others	6 (3.4%)	8 (4.7%)	16 (5%)	19 (4.4%)	
Marital status					0.044
Married	88 (49.2%)	82 (48.2%)	180 (56.6%)	250 (58.5%)	
Unmarried	91 (50.8%)	88 (51.8%)	138 (43.4%)	177 (41.5%)	
T stage					0.013
T0	4 (2.2%)	1 (0.6%)	7 (2.2%)	7 (1.6%)	
T1	27 (15.1%)	46 (27.1%)	48 (15.1%)	55 (12.9%)	
T2	54 (30.2%)	39 (22.9%)	74 (23.3%)	103 (24.1%)	
T3	36 (20.1%)	38 (22.4%)	82 (25.8%)	113 (26.5%)	
T4	58 (32.4%)	46 (27.1%)	107 (33.6%)	149 (34.9%)	
N stage					0.030
N0	48 (26.8%)	49 (28.8%)	57 (17.9%)	99 (23.2%)	
N1	21 (11.7%)	17 (10%)	27 (8.5%)	42 (9.8%)	

N2	77 (43%)	77 (45.3%)	152 (47.8%)	175 (41%)	
N3	33 (18.4%)	27 (15.9%)	82 (25.8%)	111 (26%)	
Laterality					0.582
Left	70 (39.1%)	63 (37.1%)	126 (39.6%)	172 (40.3%)	
Right	99 (55.3%)	104 (61.2%)	181 (56.9%)	237 (55.5%)	
Others	10 (5.6%)	3 (1.8%)	11 (3.5%)	18 (4.2%)	
Primary site					0.042
Main bronchus	17 (9.5%)	9 (5.3%)	27 (8.5%)	33 (7.7%)	
Upper lobe	70 (39.1%)	98 (57.6%)	157 (49.4%)	219 (51.3%)	
Middle lobe	12 (6.7%)	5 (2.9%)	12 (3.8%)	19 (4.4%)	
Lower lobe	50 (27.9%)	47 (27.6%)	83 (26.1%)	98 (23%)	
Others	30 (16.8%)	11 (6.5%)	39 (12.3%)	58 (13.6%)	
Brain Metastasis					<0.001
Yes	37 (20.7%)	117 (68.8%)	29 (9.1%)	237 (55.5%)	
No	142 (79.3%)	53 (31.2%)	289 (90.9%)	190 (44.5%)	
Bone Metastasis					0.667
Yes	56 (31.3%)	64 (37.6%)	110 (34.6%)	147 (34.4%)	
No	123 (68.7%)	106 (62.4%)	208 (65.4%)	280 (65.6%)	
Liver Metastasis					<0.001
Yes	60 (33.5%)	42 (24.7%)	145 (45.6%)	106 (24.8%)	
No	119 (66.5%)	128 (75.3%)	173 (54.4%)	321 (75.2%)	
Lung Metastasis					<0.001
Yes	48 (26.8%)	29 (17.1%)	93 (29.2%)	77 (18%)	
No	131 (73.2%)	141 (82.9%)	225 (70.8%)	350 (82%)	

Abbreviations: LCNEC: large cell neuroendocrine carcinoma.

Table S2. The baseline characteristics of different treatment modalities in IV SCLC

Variable	Control (n=2979)	Radiotherapy (n=1291)	Chemotherapy(n=8242)	Chemoradiotherapy(n=8427)	p-value
Age					<0.001
＜65	2264 (76%)	899 (69.6%)	5163 (62.6%)	4338 (51.5%)	
≥65	715 (24%)	392 (30.4%)	3079 (37.4%)	4089 (48.5%)	
Gender					<0.001
Male	1457 (48.9%)	626 (48.5%)	4349 (52.8%)	4267 (50.6%)	
Female	1522 (51.1%)	665 (51.5%)	3893 (47.2%)	4160 (49.4%)	
Race					0.005
Black	259 (8.7%)	139 (10.8%)	665 (8.1%)	797 (9.5%)	
White	2578 (86.5%)	1095 (84.8%)	7235 (87.8%)	7272 (86.3%)	
Others	142 (4.8%)	57 (4.4%)	342 (4.1%)	358 (4.2%)	
Marital status					<0.001
Married	1271 (42.7%)	574 (44.5%)	4268 (51.8%)	4659 (55.3%)	
Unmarried	1708 (57.3%)	717 (55.5%)	3974 (48.2%)	3768 (44.7%)	
T stage					<0.001
T0	46 (1.5%)	21 (1.6%)	119 (1.4%)	102 (1.2%)	
T1	337 (11.3%)	193 (14.9%)	866 (10.5%)	872 (10.3%)	
T2	735 (24.7%)	320 (24.8%)	1955 (23.7%)	1894 (22.5%)	
T3	684 (23%)	262 (20.3%)	1830 (22.2%)	1823 (21.6%)	
T4	1177 (39.5%)	495 (38.3%)	3472 (42.1%)	3736 (44.3%)	
N stage					<0.001
N0	539 (18.1%)	239 (18.5%)	870 (10.6%)	961 (11.4%)	
N1	1590 (53.4%)	646 (50%)	4492 (54.5%)	4520 (53.6%)	
N2	208 (7%)	128 (9.9%)	528 (6.4%)	540 (6.4%)	
N3	642 (21.6%)	278 (21.5%)	2352 (28.5%)	2406 (28.6%)	

Laterality					0.101
Left	1280 (43%)	513 (39.7%)	3424 (41.5%)	3460 (41.1%)	
Right	1572 (52.8%)	725 (56.2%)	4478 (54.3%)	4665 (55.4%)	
Others	127 (4.3%)	53 (4.1%)	340 (4.1%)	302 (3.6%)	
Primary site					<0.001
Main bronchus	316 (10.6%)	137 (10.6%)	980 (11.9%)	1100 (13.1%)	
Upper lobe	1364 (45.8%)	646 (50%)	3815 (46.3%)	4234 (50.2%)	
Middle lobe	114 (3.8%)	40 (3.1%)	332 (4%)	308 (3.7%)	
Lower lobe	711 (23.9%)	282 (21.8%)	1821 (22.1%)	1635 (19.4%)	
Others	474 (15.9%)	186 (14.4%)	1294 (15.7%)	1150 (13.6%)	
Brain Metastasis					<0.001
Yes	467 (15.7%)	768 (59.5%)	804 (9.8%)	3500 (41.5%)	
No	2512 (84.3%)	523 (40.5%)	7438 (90.2%)	4927 (58.5%)	
Bone Metastasis					<0.001
Yes	893 (30%)	446 (34.5%)	3269 (39.7%)	3038 (36.1%)	
No	2086 (70%)	845 (65.5%)	4973 (60.3%)	5389 (63.9%)	
Liver Metastasis					<0.001
Yes	1296 (43.5%)	383 (29.7%)	4402 (53.4%)	2714 (32.2%)	
No	1683 (56.5%)	908 (70.3%)	3840 (46.6%)	5713 (67.8%)	
Lung Metastasis					<0.001
Yes	705 (23.7%)	231 (17.9%)	1865 (22.6%)	1534 (18.2%)	
No	2274 (76.3%)	1060 (82.1%)	6377 (77.4%)	6893 (81.8%)	

Abbreviations: SCLC: small cell lung cancer.
